# Formulation and Physical–Chemical Analysis of Functional Muffin Made with Inulin, Moringa, and Cacao Adapted for Elderly People with Parkinson’s Disease

**DOI:** 10.3390/antiox13060683

**Published:** 2024-05-31

**Authors:** Paula García-Milla, Rocío Peñalver, Gema Nieto

**Affiliations:** 1 Department of Food Technology, Nutrition and Food Science, Veterinary Faculty, University of Murcia, Regional Campus of International Excellence “Campus Mare Nostrum”, Campus de Espinardo, 30100 Murcia, Spain; ppaulagm@gmail.com (P.G.-M.); rocio.penalver@um.es (R.P.); 2 Nutrition and Dietetics Program, Faculty of Health Sciences, Universidad Autónoma de Chile, Providencia 7500975, Chile

**Keywords:** muffins, fiber, inulin, constipation, Parkinson’s disease

## Abstract

Parkinson’s disease (PD) is a neurodegenerative disorder that affects people’s health. Constipation is probably one of the most prominent gastrointestinal symptoms (non-motor symptoms) of PD with devastating consequences. The aim of this research work is to formulate a functional food product, supplemented with inulin, cocoa, and Moringa, which can be an adjuvant in the treatment of constipation. The product was prepared according to a muffin or “Chilean cake” recipe; this basic muffin was prepared with additions of inulin (MI), inulin + cacao (MIC), and inulin + Moringa (MIM). A physical–chemical analysis of the macronutrients and an antioxidant capacity assessment of the samples were conducted, as well as a sensory evaluation performed by a group of people suffering from Parkinson’s disease. A statistically significant difference was observed in the soluble (*p* = 0.0023) and insoluble (*p* = 0.0015) fiber values between the control samples and all samples. Furthermore, inulin + cacao improved the antioxidant capacity and folate intake compared to the control. Inulin alone has been shown to have antioxidant capacity according to ABTS (262.5728 ± 34.74 μmol TE/g) and DPPH (9.092518 ± 10.43 μmol TE/g) assays. A sensory evaluation showed a preference for the product with inulin and for the product with inulin + cacao, with a 78% purchase intention being reported by the subjects who evaluated the products. The incorporation of inulin and cacao improved the nutritional value of the muffins; the dietary fiber, antioxidant capacity and folate content are some of the features that stood out. A bakery product enriched with inulin, cocoa and Moringa could serve as a nutritional strategy to enhance nutritional value, thus helping in the treatment of constipation.

## 1. Introduction

Parkinson’s disease (PD) is the second most prevalent neurodegenerative disease worldwide after Alzheimer’s disease [1,2].

It is estimated to affect between 1% and 2% of individuals aged over 65. In 2016, 6.1 million cases were reported, a figure that is expected to double by 2050, affecting the quality of life and health of people suffering from the disease [2].

According to the data provided by the Spanish Society of Neurology, approximately 10,000 new cases are diagnosed each year in the country, and it is estimated that around 120,000 to 150,000 people will be affected by the disease [3]. On the other hand, Chile has the highest prevalence in the Latin America region [4], with an estimated raw prevalence of 160.7/100,000 inhabitants according to a recently published study [5].

Parkinson’s disease is a complex, chronic, and progressive neurodegenerative disorder that affects dopaminergic neurons [6], producing a deficit of brain dopamine that leads to the characteristic symptoms of the disease, such as tremor, rigidity, and bradykinesia, a set of symptoms known as motor symptoms [7]. In addition, postural instability emerges as the disease progresses [8]. PD is also characterized by non-motor symptoms, such as sleep disorders, urinary problems, sialorrhea, depression, cognitive disorders, swallowing issues, sexual disorders, cardiovascular disorders, and gastrointestinal diseases, among others [9,10].

Constipation is probably one of the most prominent gastric symptoms of PD [11]; it is highly prevalent (50% to 80%) and is related to an increased risk of suffering from the disease [12,13,14]. Constipation has also been identified as an important prodromal symptom that may precede the classic symptoms of PD by decades, occurring many years before a person is even diagnosed [15,16].

Constipation has devastating consequences for people with PD, affecting quality of life and health. According to a recent study, constipation is associated with increased cognitive impairment, even serving as a marker to identify neurocognitive disorders in patients with PD [17]. Additionally, it can play important roles in motor fluctuations and in the absorption of levodopa (the gold standard treatment for the disease), which can affect levodopa absorption and efficacy [18,19].

The general therapeutic approach for constipation consists of eating a diet high in fruits, vegetables, and whole grain cereals, an adequate intake of water, in addition to an active lifestyle. However, overall patterns are often insufficient in patients with PD since this condition may be part of the disease process in which the autonomic nervous system is affected, causing the movements of the gastrointestinal tract to slow down [20,21].

For this reason, a large proportion of patients suffering from the disease require the incorporation of laxatives or dietary fiber supplements in addition to general measures.

Inulin (a chicory-derived prebiotic fiber) has been shown to have beneficial effects on health and intestinal transit; moreover, it might alter gut microbiota [22]. This was demonstrated by a systematic review where the effect of inulin on the intestinal microbiota was evaluated, and it was found that a supplementation of 5 to 20 g per day increased the relative abundance of *Anaerostipes*, *Faecalibacterium*, and *Lactobacillus* and resulted in a relative decrease in *Bacteroides* [23]. In addition, it was found that antioxidants could play a fundamental role in intestinal health, as well as in other diseases, such as Parkinson’s disease [24].

On the other hand, a randomized clinical trial evaluating the effect of incorporating inulin in apple juice regarding stools found that the consumption of juice supplemented with inulin significantly increased (*p* ≤ 0.05) the frequency of stools [25].

This undoubtedly makes inulin much more interesting from a therapeutic point of view.

Given the relevance that the management of constipation has to people with Parkinson’s disease, the objective of this research work is to formulate a food product rich in inulin-based prebiotic dietary fiber that allows for the development of innovative therapeutic strategies to address constipation in elderly people with Parkinson’s disease.

## 2. Materials and Methods

We conducted a physical and chemical analysis of a food intended for elderly people with Parkinson’s disease.

This research has not been submitted for evaluation by the ethics committee since it does not involve intervention in people or animals, and consequently, informed consent is not required.

### 2.1. Preparation of the Product

For the preparation of the product, we decided to make an easy-to-prepare food using easily accessible ingredients; for this reason, a recipe for a muffin or “basic Chilean cake” was developed.

The control food product was prepared by mixing the dry ingredients: 150 g of white wheat flour, 5 g of baking powder, and 100 g of sugar. Separately, one egg white was beaten until stiff peaks formed; then, an egg yolk (1 unit), 50 g of olive oil, and 115 g milk of were added. All of these ingredients were carefully mixed until a smooth batter was obtained.

For cooking, the oven was preheated to 180 °C; individual molds for 6 servings were used, and the muffins were baked for 30 min.

In the case of the fiber-supplemented muffin, 30 g of chicory-derived inulin was added for every 150 g of white wheat flour; inulin was incorporated as a substitute for 60% of the fat portion provided by olive oil, that is, 30 g of inulin and 20 g of olive oil were used. The same procedure and ingredients used for the preparation of the control product were used to prepare this muffin. In addition, different flavors were produced by incorporating Moringa oleifera and cacao to the batter supplemented with inulin in order to obtain additional therapeutic properties with the aim of developing a therapeutic and functional product (Table 1).

### 2.2. Physical–Chemical Analysis

#### 2.2.1. Determination of Macronutrients

##### Determination of Moisture

The oven drying method (AOAC, 1995) was used to determine the sample moisture content; 3 g of sample was weighed on a precision balance inside an appropriately labeled Petri dish. Subsequently, the sample was left to dry at 110 °C in a forced-air oven for 24 h [26,27].

The obtained results are expressed as percentages, and the following calculations were performed:

Dry matter %: 100 × (Fw − Ew)/Sw Moisture %: 100 − Dry matter

where Fw is the final weight of the dish; Ew is the empty dish weight; and Sw is the sample weight.

##### Determination of Inorganic Matter

The technique specified in the AOAC method 923.03 (1990) was used, which is based on the complete incineration of the inorganic matter in the sample using a muffle furnace at 525 °C, with only the residue of inorganic matter being left [27].

An appropriately labeled crucible was used for the procedure; 1 g of fresh sample was weighed and placed on a heating plate to start combustion. Once the sample bulk had been reduced, it was placed inside a muffle furnace at 525 °C until pure white ash was obtained without remains of organic matter.

The following formula was used for percentage determination:

Ashes %: ((W2 − W0)/(W1 − W0)) × 100

where W0 is the weight of the empty crucible; W1 is the weight of the crucible plus the sample; and W2 is the weight of the crucible plus ash.

##### Determination of Energy Value

The energy value of the samples was obtained by adding the energy values of proteins, carbohydrates, and fats of each sample.

For this purpose, the following Atwater conversion values (WHO, 1985) were used [26,28]:

Protein: 4 calories per gram; carbohydrates: 4 calories per gram; fats: 9 calories per gram.

##### Determination of Total Nitrogen and Protein Content

The Kjeldahl procedure, described in the AOAC method 955.04 (1990) [24], was used for the determination of the total protein content of the sample.

This method is based on the destruction of organic matter using concentrated sulfuric acid, thus forming ammonium sulfate. When an excess of sodium hydroxide is added, ammonia is released, which is distilled over boric acid, forming ammonium borate, which is titrated with hydrochloric acid.

An amount of 2 g of fresh sample was weighed, and 1 catalyst tablet plus 15 mL of concentrated sulfuric acid were added. After that, the tubes were placed in a heating block and digested in stages until they reached 450 °C, maintaining the temperature for 1 h. After time had passed, they were left to cool down, and distillation was carried out with the addition of 38% NaOH until a color change occurred as a consequence of a change in pH.

Subsequently, distillate titration with 0.1 N of HCl was performed to determine the amount of ammonia absorbed by boric acid.

The following expression was used for the calculation of proteins:

Crude protein %: ((V2 − V1) × 0.1)/W × 1.4 × F

where V1 is the volume in mL of the required HCl solution; V2 is the volume in mL of HCl required for the sample; normality is considered at 0.1; W is the weight of the sample in grams; and F is the conversion factor as follows:

6.25: meat, fish, eggs, legumes, and vegetable protein.
5.7: cereals and soy derivatives.
6.38: milk and dairy products.
5.55: jelly.
5.95: rice.

##### Determination of Carbohydrates (Carbs)

The determination of carbs was carried out according to the recommendation of the FAO/WHO (1982) in which carbohydrates are calculated by determining the difference based on the sum of fat (F), ash (A), protein (P), moisture (M), and dietary fiber (DF), according to the following expression [26,29]:

Total carbs (%): 100 − (F + A + P + M + DF) Available carbs (%): Total carbs − DF

##### Determination of Total Fat

For the determination of total fat, the procedure described by the AOAC 1990 method [24] was used, and a Tecator Soxhlet-type extractor was used.

One gram of sample, which was previously dried for the determination of moisture, was weighed and placed into the fat extractor using the metal ring. The sample was properly handled by using gloves and tweezers.

The aluminum cups were dried, weighed, and appropriately labeled, and then they were transported in a desiccator to avoid weight interference by moisture.

Once the cups and samples were assembled, 50 mL of ether was added to each of them, and the procedure, which lasted 2 h, was performed. After time had passed, the aluminum cups were transported to the oven to be dried for at least 2 h, thus eliminating ether residues contained in the cups. Once the samples were cooled down, they were weighed, and the fat value was determined according to the following equation:

Fat (%): ((W1 − W2)/W) × 100

where W1 is the weight of the cup with ether extract or fat residue; W2 is the weight of the empty cup; and W is the weight of the sample.

#### 2.2.2. Determination of Folate Content

The folate content was determined according to the protocol described by Peñalver, R (2023) [30], which is based on the protocol by Konings et al. (1999) and Pfeiffer et al. (1997) [31,32,33].

An amount of 1 g of the sample was weighed and combined with 25 mL of extraction buffer (50 mmol/L, 50 mmol/L HEPES, containing 2 g of sodium ascorbate/100 mL and 10 mmol/L 2-mercaptoethanol, pH 7.85) under a nitrogen atmosphere. The mixtures were then placed in a boiling water bath for 10 min, followed by cooling and homogenization. Subsequently, the pH was adjusted to 4.9 using 60 mmol/L HCl.

The enzymatic deconjugation and purification of samples were carried out according to the method outlined by Peñalver et al. [30], which is based on the method by Vahteristo et al. (1996) [34]. An aliquot of 5 mL was incubated for 3 h at 37 °C under a nitrogen atmosphere with 1 mL of hog kidney conjugase and 1 mL of α-amylase preparation (20 mg/mL in 1 g sodium ascorbate/100 mL). The samples were then boiled at 100 °C for 10 min to deactivate the enzymes.

After cooling, the samples were filtered through a 0.45 μm pore size and purified using strong anion exchange (SAX) cartridges connected to a Supelco 12-port vacuum manifold. Initially, the cartridges were conditioned with 3 mL each of n-hexane, methanol, and Mili-Q water and then equilibrated with 3 mL of purification buffer (10 mmol/L dipotassium hydrogen phosphate, 2-mercaptoethanol (v/v), pH 7.0). Subsequently, the samples were slowly loaded and eluted with 2 mL of elution buffer (10 g of sodium chloride/100 mL, 10 mmol/L of sodium acetate, 1 g of ascorbic acid/100 mL) at a flow rate of <0.5 mL/min. The eluted samples were then weighed.

Sample analysis was performed using HPLC/MS; a mass spectrometer was operated in positive mode. The nebulizer gas pressure was set to 40 psi, while the drying gas flow was set at 16 L/min at a temperature of 150 °C, and the sheath gas flow was set at 12 L/min at a temperature of 300 °C. The capillary spray, nozzle, fragmentor, and octopole 1 RF Vpp voltages were set to 4000 V, 1000 V, 350 V, and 750 V, respectively. Profile data in the 100–600 m/z range were acquired for MS scans in 2 GHz extended dynamic range mode with 3 spectra/s, 333.3 ms/spectrum, and 1999 transients/spectrum. A reference mass of 121.0509 was used for mass correction during analysis.

Data analysis was conducted with the MassHunter Qualitative Analysis Navigator software (Agilent Technologies, Rev. B.08.00) [27].

#### 2.2.3. Determination of Dietary Fiber

For the determination of total dietary fiber, the AOAC 985.29 method (1990) described by Prosky et al. (1985) [35] was used.

For digestion, 1 g of the sample was weighed and 50 mL of phosphate buffer (0.05 M, pH 6) was added; afterwards, the samples were taken to a bath at 95 °C, and 0.2 mL of α-amylase was added to gelatinize the starch. After 30 min, the samples were cooled to room temperature and the pH was adjusted to 7.5.

The samples were then transferred to a 60 °C bath, and 1 mg of protease was added to remove proteins; to this end, samples were agitated for 30 min.

After time had passed, samples were cooled down and pH was adjusted to 4.5, and 0.3 mL of amyloglucosidase enzyme was added to reheat in a bath at 60 °C for 30 min.

Sample filtration: The volume of the previously described digestion mixture included the addition of 280 mL of 95% ethanol at 60 °C, leaving the fiber to precipitate for at least 1 h; then, the samples were filtered using a Fibertec™ 1023 (FOSS analyzer, Denmark) in which a crucible containing 0.5 g of Celite was placed. At the end of the filtration process, the residue was left in the oven, and once dried, it was weighed, and crucibles were taken out to determine the protein and ash contents.

#### 2.2.4. Determination of Antioxidant Capacity

Four techniques were performed to assess the antioxidant capacity of the muffin enriched with prebiotic fiber. The ORAC, FRAP, ABTS, and DPPH techniques were used, which are described below.

##### Oxygen Radical Absorbance Capacity (ORAC) Technique

This method was implemented following the procedure outlined by Ehlenfeldt and Prior (2001) [36]. A 96-well black plate was utilized, with the edges filled with 200 μL of distilled water; blank samples consisted of 20 μL of phosphate buffer (utilized for sample dilution) and 20 μL of samples and Trolox standards. Once the plate was prepared, the procedure commenced in the Synergy HT plate reader after flushing with water and the appropriate reagents. The GEN 5 program was employed, wherein the protocol was set as follows: 200 μL of fluorescein was placed in each well; after 15 min, 20 μL of AAPH (0.216 g in 10 mL of phosphate buffer) was added to each well and subsequently measured with excitation at 485 nm and emission at 528 nm every minute for 90 min. The entire process was carried out at 37 °C. Supportive software was used to calculate the area under the curve for all measurements, and the data were extrapolated using Trolox standard curves (of known concentrations) [27].

##### Ferric Reducing Antioxidant Power (FRAP) Technique

For the ferric reducing antioxidant power (FRAP) assay, we used the protocol described by Benzie and Strain (1999) [37]. A FRAP solution consisting of 20 mL of acetate buffer, 2 mL of TPTZ, and 2 mL of FeCl_3_·6H_2_O was prepared. In the cuvette, 1 mL of FRAP solution and 100 μL of the sample were added; readings were conducted at an absorbance of 593 nm for 4 min. FRAP solution was used for the blank. Finally, a standard Trolox curve was generated to compare the results.

##### ABTS Technique

This technique assesses the ability of antioxidant compounds to capture the radical cation that is generated after the reaction between 2,2′-azino-bis (3-ethylbenzothiazoline-6-sulphonic acid) (ABTS) and MnO_2_ [27].

The method used was that described by Re et al. (1999) [38]. Together, 1 mL of ABTS solution and 100 μg of the sample were used; the mixture was shaken for 30 s, and absorbance was read at 734 nm after 2 min. For the blank, an ABTS solution with an absorbance of approximately 0.7 was used, which was adjusted with water and MnO_2_.

##### DPPH Technique

This method was derived from the procedures outlined by Brand-Williams et al. (1995) and Sánchez-Moreno et al. (1998) [39,40]. The DPPH reagent was prepared by dissolving 0.0063 g of DPPH in 250 mL of ethanol, and it was shielded from light. A mixture comprising 3.9 mL of DPPH reagent and 100 μL of sample and standard solution was prepared; absorbance was recorded at 515 nm after 30 min of incubation (ensuring the preparation was shielded from light). The baseline was established with methanol, and the blank consisted of the DPPH reagent without any samples.

### 2.3. Sensory Analysis and Product Tasting

#### 2.3.1. Colorimetry

In order to determine the color of the product, researchers used a tristimulus colorimeter, that is, a tool that uses three filters to measure the colors red, green, and blue. The place was equipped with a light-colored table on which the samples were placed on an opaque white paper; the room light conditions corresponded to natural lighting conditions.

**Figure 1:**
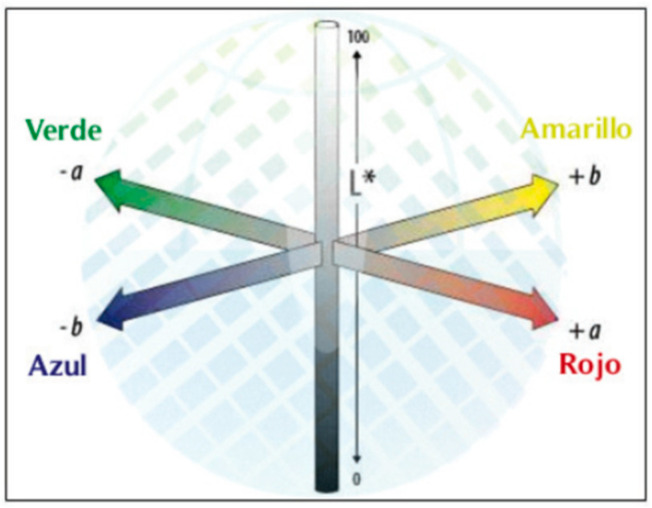
The color coordinates in the Hunter system [41].

Color measurement was conducted based on the color coordinates of the Hunter color system (Figure 1) [41].

#### 2.3.2. Degustation of the Product (Product Tasting)

Product tasting was carried out in the VITALIS Research Center at the University of Murcia, Spain.

**Figure 2:**
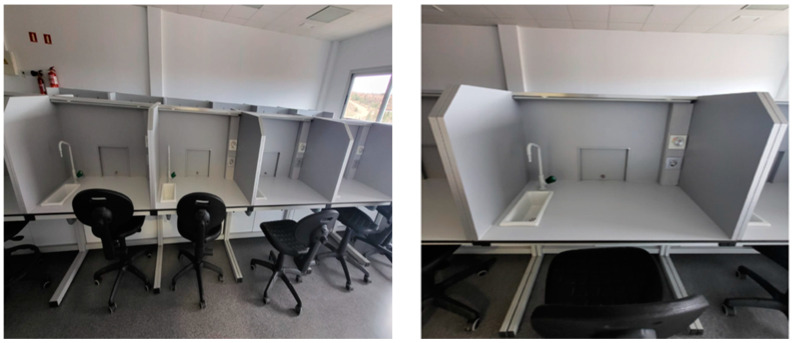
Professional room for performance of product tasting, University of Murcia, Spain.

Room conditions were adjusted in accordance with the UNE-EN ISO 8589:2010 [42]. Tasting tests were conducted in a room with light-colored smooth walls and furniture under natural lighting (Figure 2). Conditions of temperature and humidity were comfortable (22 °C and 60–70%). Each individual booth was provided with a glass of water, and the product was presented on a white disposable plate; white napkins, a pen, and a printed version of the assessment document were also included. Individual booths avoided distractions and did not allow communication between the subjects, who were separated from each other [43]. A group of people from the Parkinson’s Association of Murcia were invited to participate in the study as evaluation judges.

The studied attributes were evaluated using a five-point hedonic scale in which the panelists gave a score ranging from 1 (dislike very much) to 5 (like very much). The assessed attributes corresponded to appearance, aroma, texture, taste, color, purchase intention, and overall acceptability.

### 2.4. Statistical Analysis

Statistical analysis was performed using the statistical software for data science STATA version 16. The ANOVA parametric test was used for the analysis of variance, and the Bonferroni test was used for multiple comparisons, with *p* < 0.005 representing statistical significance.

## 3. Results and Discussion

### 3.1. Composition of the Product and Its Benefits

The food industry has mainly incorporated inulin in products such as cookies, sponge cakes, ice cream, and other dairy products such as yogurts and desserts.

Most of the uses described above are based on its function as a thickener, emulsifier, and gelling agent, and it can be used as a substitute for sugar and/or fats [44], modifying the nutritional value of the final product.

This project was based on the guidelines by Rodríguez J (2014), who recommended substituting fat with inulin in bakery products, particularly sponge cakes [45]. According to published studies, fat can be replaced by inulin in a percentage ranging from 19% to 100% [46,47]. In our study, we performed initial tests that included the preparation of products with the addition of 40%, 50%, 60%, and 70% inulin. After a tasting test conducted by a group of experts, it was determined that the best concentration was 60% inulin since it did not alter the sensory characteristics, especially the texture perceived on the palate. As more inulin is added, the texture is perceived as “harder” or “gummier” due to the formation of gels produced by the incorporation of inulin.

In addition to the inulin-enriched version, we developed two products including food ingredients that have nutritional and medicinal properties due to their antioxidant activity; to this end, Moringa and cacao were used, as shown in Table 1.

With respect to the selection of products, we opted for cacao because of its taste and the overall acceptability that it has in pastry and bakery products, as well as its attributes and health benefits.

A research study found that cacao has a high content of total dietary fiber (60.54 ± 0.32) with a predominance of insoluble fiber (50.29 ± 0.70), thereby positioning cacao as an excellent ingredient for the development of dietary fiber-enriched functional foods [47]. In relation to its antioxidant capacity, unfermented cacao bean has 1.98 ± 0.04 mmol of Trolox/g versus dark chocolate (fermented), which has 0.39 ± 0.01 mmol of Trolox/g, while the total polyphenol values experience the same variations, going from 34.80 ± 3.10 to 6.37 ± 0.09, respectively, according to the analysis conducted by Tafurt et al. [48]. Furthermore, the antioxidant capacity of cacao and its properties attributable to phytonutrients have been associated with beneficial effects in several diseases, including cardiovascular and neurodegenerative diseases such as Alzheimer’s disease and Parkinson’s disease [49,50].

Moringa has been shown to have therapeutic properties, just as cacao, among which we may mention health benefits for individuals affected by neurological diseases, diabetes mellitus, cancer, and cardiovascular disease [51,52,53].

Its nutritional composition provides 25.30 ± 0.27 g of protein/100 g; 5.75 ± 0.21 g of fat/100 g; 29.80 ± 4.55 g of carbohydrates/100 g; and 24.97 ± 4.55 g of dietary fiber/100 g [54]. In addition, it has a significant antioxidant activity, with a total phenolic content of 32.90 ± 4.38 mg of GAE/g, which is a property derived from the presence of ascorbic acid, flavonoids, phenolics, and carotenoids [51].

### 3.2. Determination of Macronutrients

Table 2 shows a proximate analysis of the following products: the control, whose preparation is based on a basic Chilean muffin recipe (Control); the sample enriched with inulin, which includes 30 g of inulin in 150 g of flour (MI); the sample enriched with inulin and cacao (MIC); and finally, the sample enriched with inulin and Moringa (MIM).

According to the obtained results, we found that the product enriched with inulin has a lower caloric value compared to the control, with the sample enriched with cacao having the lowest total caloric value. Nevertheless, the differences observed in the total caloric value are not statistically significant, as well as the differences found in the protein, ash, and carbohydrate contents.

However, regarding the moisture content of the product, we can assure that it varied with respect to the control, with increased moisture being observed, particularly in the Moringa sample, with a statistically significant difference (*p* = 0.0016). In addition, a significant change was found in the fat content of the product (*p* = 0.0078), which decreased with respect to the control; these values are not a surprise given the composition of the product (Table 2).

According to Rodríguez JP (2016), a reduction of 22 calories would be obtained by substituting fat with 45% inulin; this is equivalent to 36% less fat and a 14% lower total caloric value. In our study, we found an average calorie reduction of 62 kcal, which corresponded to 17% of the total caloric value, and regarding fat contribution, we obtained an average reduction of 8.81 g, corresponding to 47%. Our results are similar to those obtained by Rodríguez JP, who achieved a fat reduction of 45%, going from 4 g to 2.4 g [55]. These findings are also in line with the study by Doménech G et al. (2016), where inulin was used as a substitute for fat, and statistically significant reductions in calories and fat were obtained in all of the products prepared, which included cookies, croissants, muffins, and sponge cakes [56].

Regarding protein intake, it should be noted that people with Parkinson’s disease experience a significant drug–nutrient interaction (protein–levodopa), which is why it is considered that high-protein diets could exacerbate the symptoms of the disease [57]. To avoid this complication in the treatment of PD, low-protein diets or protein redistribution (consuming the majority of protein intake at dinner) have been proposed [58]; however, these strategies are not exempt from complications. It is important to note that there is a high prevalence of malnutrition and sarcopenia in people with PD, and even more so among elderly people [59], so low-protein diets can pose risks if maintained in the long term.

A protein intake of around 6–7 g (value provided by the muffin), delivered in a snack, administered at least 40–60 min apart from levodopa, could be a nutritional strategy to maintain muscle mass and improve the health of people with Parkinson’s disease.

### 3.3. Determination of Dietary Fiber

The assessment of dietary fiber revealed an increase in fiber when comparing the control and the product supplemented with inulin (MI) in all the analyses (soluble fiber, insoluble fiber, and total fiber). Nevertheless, the products with additions of cacao and Moringa did not show an increased fiber content compared to the control (Table 3).

The statistical analysis (ANOVA) yielded statistically significant differences in insoluble fiber intake (*p* = 0.0015) and soluble fiber intake (*p* = 0.0023) when comparing the control product with the product including the addition of inulin (MI) (Table 3).

In general, studies that have incorporated inulin in bakery products (cookies, sponge cakes, brownies, etc.) mainly focused on the characteristics that inulin gives to the dough, as well as on the ability of inulin to achieve fat reduction in the final product [60,61]. However, it is very difficult to find analyses focused on the contribution of dietary fiber despite the fact that all studies address this matter.

Despite this limitation, we found a very interesting article that indicates that the loss of fructans due to the baking process in bakery products ranges from 35% to 47% depending on the harvest of the product, as well as the companies that formulate the inulin derived from Jerusalem artichokes, while the production of fructose after hydrolysis ranges from 11% to 45.8% [62].

It should be noted that inulin is a polysaccharide composed of fructo-oligosaccharides, and it is considered an important natural fructan and a soluble fiber par excellence [63]. The health benefits of inulin have been widely studied, especially those related to cardiovascular health. These benefits were demonstrated in a randomized clinical trial including 34 subjects who were given inulin-enriched cookies for 1 month. The obtained results show a significant reduction in the total cholesterol levels (223.1 ± 45.3 mg/dL vs. 208.8 ± 33.1 mg/dL; *p* < 0.05) and LDL cholesterol levels (142.9 ± 39.2 mg/dL vs. 131.4 ± 28.6 mg/dL; *p* < 0.05), concluding that 3 g of inulin from enriched cookies reduced cholesterol in subjects with obesity [64]. In addition to its benefits in cardiovascular health, its effect on the gut microbiota (bacteria living in the gut that confer a health benefit) has been widely discussed, as demonstrated by a randomized, double-blind, placebo-controlled, cross-over study in 34 healthy participants, who were classified as high-fiber consumers and low-fiber consumers. The results show that the group that underwent intervention with prebiotic fiber had an increase in Bifidobacterium (*p* = 0.001), concluding that the subjects who consumed high levels of fiber had a greater gut microbiota response and were more likely to benefit from the supplementation of inulin-type fructans than those with low fiber intakes [65].

Inulin can also be beneficial for brain health, as proposed by Bizeau JB et al. (2022) [66], who conducted a study in 5-week-old male C57BL/6J mice who were fed a diet supplement with chicory-derived inulin for 11 weeks.

It is important to note that the lipid content in the brain is crucial for its health; an imbalance in brain lipids has been implicated in the occurrence of neurodegenerative diseases such as Alzheimer’s disease and Parkinson’s disease.

The researchers found that inulin supplementation did not alter total plasmalogen (glycerophospholipids) in the brain cortex of mice; however, it affected the brain plasmalogen levels of ethanolamine species, which represent a significant reservoir of DHA. Despite these results, researchers stated that inulin may have deleterious effects and that the real mechanism that links inulin with gut microbiota and brain plasmalogen levels has not been fully demonstrated [66].

The health benefits of inulin make it an excellent functional nutritional food for individuals affected by Parkinson’s disease.

### 3.4. Determination of Antioxidant Activity

According to the analysis of variance, it was found that, in general, the antioxidant capacity of the products was improved with the addition of cacao and Moringa. However, of the four assays performed (DPPH, ABTS, ORAC, and FRAP), only the ORAC and FRAP assays showed a statistically significant difference (*p* = 0.000), as shown in Table 4.

When comparing the samples (Table 5), it can be noticed, specifically in the ORAC assay, that there is a statistically significant difference between the control and the MI (basic muffin with inulin) and the MIC (muffin with inulin and cacao) samples. In addition, it is observed that the MIC has a higher antioxidant capacity as compared to the MI and MIM (muffin with inulin and Moringa) samples (*p* < 0.005). While when the FRAP assay was conducted, it was found that the MIC has a higher antioxidant capacity than the control, the MI and the MIM (*p* < 0.005).

Inulin is known to be a non-digestible carbohydrate, and its benefits are recognized because of its effect on the gut; however, there has been little discussion on its antioxidant activity. In this study, the antioxidant activity of inulin was analyzed by conducting ABTS and DPPH assays. It was found that inulin has an antioxidant capacity, which, although it may be low, exists and provides the final product with an added health value. As shown in Table 6, inulin has a value of 262.5728 ± 34.74 μmol TE/g, as found in the ABTS assay, and a value of 9.092518 ± 10.43 μmol TE/g according to the DPPH assay.

Table 7 shows the determination of phenolic compounds. It can be noted that all of the products, including the inulin-enriched muffins, the cacao muffins, and the Moringa muffins, have a higher content of phenolic compounds compared to the control (*p* = 0.0000), with the cacao muffin (MIC) having the highest content of phenolic compounds.

### 3.5. Folate Content

Table 8 indicates the concentrations of the four folate monoglutamates as well as the total folate monoglutamates and the total folate concentrations in the three samples and control.

An increase in the folate concentration was observed in the samples enriched with inulin, cacao, and Moringa compared to the control (*p* = 0.0000). The main form of naturally occurring folate found in all samples was 5F-THF (methylfolate), and it was higher in the sample enriched with inulin + cacao (MIC). Additionally, the cacao sample had a higher total folate content in relation to all samples and the control (*p* = 0.0000).

Wheat flour has been used as a fortification vehicle for folic acid in several countries, constituting a public health strategy that allows for the reduction in health problems derived from the insufficient intake of this nutrient [67,68,69,70]. In Chile, Decree 1934 was enacted in 1951, which required mills to fortify flour with iron, calcium, and B complex vitamins. Subsequently, in 1999, this regulation was modified, obliging mills to add 2.2 mg/kg of folic acid to wheat flour [71,72]. According to an analysis published in the book by the Pan American Health Organization (PAHO) titled Nutrition and an Active Life: From Knowledge to Action, where 1 kg of bread was purchased at 50 different bakeries in the Metropolitan Region of Chile, it was determined that the folate content for 100 bread samples was 202 ± 94 μg/100 g of product. Only 9 out of 100 samples contained less than 37 μg of folic acid/100 g, to which researchers concluded that these 9 bread samples were made from unfortified flour [73].

In a study in which he analyzed bakery products, cereals, and legumes, Müller H found similar results or results well below those shown in the analysis of breads in Chile, finding that bakery products had a folate content of 14 µg/100 g in the case of those made from rye flour (whole grain rye bread), and 88 µg/100 g in the case of crispbread [74].

It should be noted that the products analyzed in this study were made from wheat flour purchased at a market in the city of Murcia in Spain, where flour is probably not fortified with folic acid, yet the product enriched with inulin + cacao (54.39 ± 0.98) obtained the highest folic acid values compared to the control and the other products tested (*p* = 0.0000).

Although some studies indicate that folic acid in fortified products is 1.7 times more bioavailable than food folate [73], the production processes of bakery products may affect the total value of folate content and each of its folate monoglutamates due to factors such as pH, temperature, humidity, etc. [75]. This was demonstrated by an analysis conducted in Uruguay, where the folic acid content of fortified flour and of a type of bread common in the country was evaluated. The results showed an average of 2.3 mg of folic acid/kg for fortified wheat flour, while “French bread” was shown to have an average of 1.7 mg/kg [76]. In order to remedy the problems related to bioavailability, the microencapsulation of nutrients can be carried out, which appears to be a safe alternative in fortified foods [77]. Nevertheless, despite the limitations, fortification with food or agri-food waste is positively correlated with the increased phenolic content and antioxidant capacity of fortified foods [78,79,80].

### 3.6. Sensory Analysis

#### 3.6.1. Colorimetry

**Figure 3:**
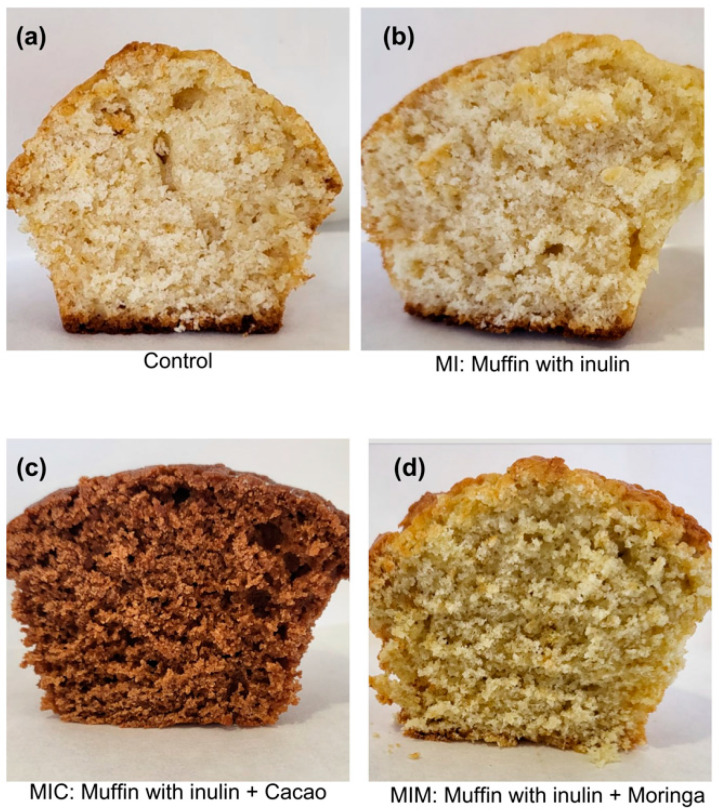
Shades of the analyzed products. (**a**) Control sample made with the basic recipe without including inulin, cocoa or moringa. (**b**) Sample prepared with inulin (MI). (**c**) Sample prepared with inulin and cocoa (MIC). (**d**) Sample made with inulin and Moringa.

A color analysis of the developed products is shown in Table 9; a statistically significant difference is observed in all color analyses. It can be seen that the control had greater lightness, total color, and presence of yellow shades; however, the product enriched with inulin differed slightly from the control, with a similar color being perceived on the two products. The product with the lowest lightness corresponded to the cacao product, which was foreseeable; in addition, this sample had a greater number of green shades and a lower saturation. Figure 3 shows the shades of the products analyzed.

#### 3.6.2. Product Tasting

A group of people with Parkinson’s disease from the Murcia community were invited to participate in the sensory analysis of the product. The sensory evaluation procedure involved 23 people whose average age was 59.1 ± 18 years old, including 11 men (48%) and 12 women (52%). Table 10 shows a descriptive analysis of the sensory evaluation. The evaluation was conducted according to a five-point hedonic scale, where a value of 5 meant “like very much” and 1 meant “dislike very much”.

**Figure 4:**
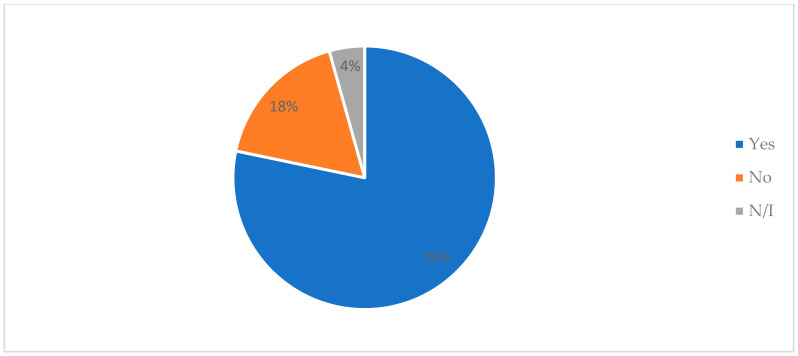
Descriptive analysis of purchase intention towards product (n = 23). N/I: non-informed.

**Figure 5:**
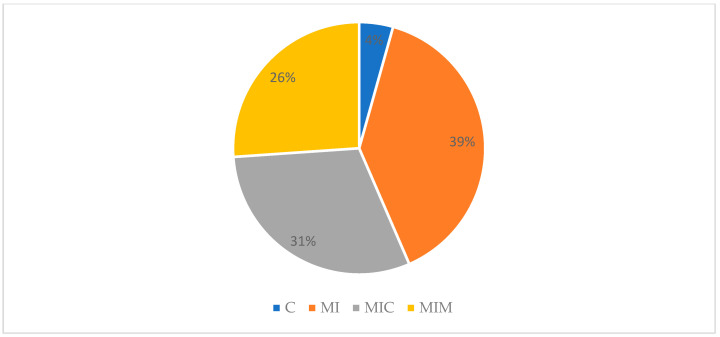
A descriptive analysis of the preference expressed by the evaluation judges. C: control; MI: inulin product; MIC: inulin product + cacao; MIM: inulin product + Moringa.

As we can see, color was generally given a value of 4 or close to 4 for all samples during the sensory evaluation; similar values were found with respect to appearance. Then, the items’ aromas, textures, overall acceptability, and purchase intentions were given a score below 4 but above 3.13 ± 1.25. It should be noted that, when asked about purchase intention towards the products presented, 78% of participants stated that they would buy the product, followed by 18% who reported that they would not (Figure 4). According to the preferences of the judges, the product of choice was the sample enriched with 39% inulin (MI), followed by the sample enriched with 31% inulin + cacao (MIC) and the sample enriched with 26% inulin + Moringa (MIM); only 4% of the participants preferred the control (Figure 5).

#### 3.6.3. The Limitations of This Study

In this study, a separate analysis of the ingredients was not carried out prior to the preparation of the final product. We believe that this is a limitation that needs to be corrected in the future.

## 4. Conclusions

People suffering from Parkinson’s disease have specific nutritional deficiencies; moreover, they experience needs that are exacerbated by the clinical progression of the disease, with the contribution of dietary fiber being one of these needs. Dietary fiber supports gut health and gut microbiota, thus promoting overall health.

This study found that adding inulin to a bakery product resulted in an improvement in the nutritional quality and soluble fiber content. Furthermore, the proposed recipe does not increase protein intake, keeping it below 10 g, which makes it very manageable with respect to the pharmacological interaction that occurs with the main drug used in the treatment of the disease.

Additionally, the antioxidant activity of the products is enhanced in the presence of inulin, with the cacao product having the highest antioxidant activity. It is important to consider food cooking processes to improve both the antioxidant capacity and bioavailability of nutrients, including folates, which were also found in greater amounts in the cacao product. Within the inulin-enriched products, the cacao product was shown to have a greater nutritional value compared to the control and the other samples with the addition of inulin. In addition, it had high acceptability among the subjects who evaluated the products, being the second most preferred product.

## Figures and Tables

**Table 1:** Sample formulation (n = 4).

Samples	Flour (g)	Baking Powder (g)	Sugar (g)	Eggs (units)	Semi-Skimmed Milk (g)	Olive Oil (g)	Inulin (g)	Cacao (g)	Moringa (ppm)
**Control**	150	5	100	1	115	50	0	0	0
**MI**	150	5	100	1	115	20	30	0	0
**MIC**	150	5	100	1	115	20	30	15	0
**MIM**	150	5	100	1	115	20	30	0	7000

MI: muffin enriched with inulin fiber; MIC: muffin enriched with inulin fiber + cacao; MIM: muffin enriched with inulin fiber + Moringa.

**Table 2:** Nutritional composition of samples (n = 4) (grams/100 g).

Parameters	Samples				
	Control	MI	MIC	MIM	ANOVA (*p* Value)
**Moisture**	23.44 ± 0.16	23.55 ± 0.25	22.47 ± 0.31	24.43 ± 0.61	*p* = 0.0016
**Energy**	367.02 ± 13.60	339.91 ± 4.06	264.42 ± 69.99	310.38 ± 14.56	*p* = 0.1647
**Protein**	6.48 ± 0.19	7.08 ± 0.55	7.36 ± 0.17	7.41 ± 0.02	*p* = 0.1054
**Carbohydrates**	43.23 ± 4.67	46.53 ± 0.53	43.59 ± 18.14	52.38 ± 0.16	*p* = 0.7562
**Fat**	18.69 ± 3.67	13.94 ± 0.45	7.77 ± 1.80	7.91 ± 1.55	*p* = 0.0078
**Ash**	0.93 ± 0.13	1.15 ± 0.11	9.09 ± 0.14.02	0.99 ± 0.15	*p* = 0.6685

Control: basic Chilean muffin; MI: muffin with inulin as fat substitute; MIC: muffin with inulin as fat substitute + cacao; MIM: muffin with inulin as fat substitute + Moringa.

**Table 3:** Determination of fiber in samples (n = 4) (grams/100 g).

Parameters	Samples				
	Control	MI	MIC	MIM	ANOVA (*p* Value)
**Insoluble Fiber**	0.87 ± 0.41	0.95 ± 0.05	5.61 ± 0.53	0.57 ± 0.75	*p* = 0.0015
**Soluble Fiber**	6.35 ± 0.68	6.80 ± 0.18	1.05 ± 0.08	5.97 ± 1.04	*p* = 0.0023
**Total Fiber**	7.22 ± 1.09	7.75 ± 0.22	6.66 ± 0.60	6.54 ± 1.79	*p* = 0.6955

Control: basic Chilean muffin; MI: muffin with inulin as fat substitute; MIC: muffin with inulin as fat substitute + cacao; MIM: muffin with inulin as fat substitute + Moringa.

**Table 4:** An intergroup analysis of the antioxidant activity of the samples (n = 4) measured (%) using the ABTS, DPPH, and μmol TE/g in ORAC and FRAP.

	Assay			
Samples	DPPH (%)	ABTS (%)	ORAC (μmol TE/g)	FRAP (μmol TE/g)
**Control**	579.92 ± 33.03	2446.39 ± 723.34	44.58 ± 6.97	2.16 ± 0.28
**MI**	661.14 ± 104.15	2835.81 ± 723.34	67.92 ± 5.84	3.18 ± 0.10
**MIC**	1844 ± 543.05	5515 ± 2050.84	99.99 ± 0.25	20.99 ± 4.09
**MIM**	548.04 ± 55.79	2407.16 ± 437.70	59.19 ± 1.43	4.66 ± 1.29
**ANOVA** **(*p* Value)**	*p* = 0.0244	*p* = 0.1419	*p* = 0.0000	*p* = 0.0000

Control: basic Chilean muffin; MI: muffin with inulin as fat substitute; MIC: muffin with inulin as fat substitute + cacao; MIM: muffin with inulin as fat substitute + Moringa.

**Table 5:** Comparative analysis between samples of antioxidant activity using the Bonferroni test.

Row Mean-Col. Mean	ORAC (μmol TE/g)			FRAP (μmol TE/g)		
	Control	MI	MIC	Control	MI	MIC
**MI**	23.34 **			1.02		
**MIC**	55.41 **	32.06 **		18.83 **	17.81 **	
**MIM**	14.61	−8.73	−40.79 **	2.50	1.48	−16.33 **

Control: basic Chilean muffin; MI: muffin with inulin as fat substitute; MIC: muffin with inulin as fat substitute + cacao; MIM: muffin with inulin as fat substitute + Moringa. Bonferroni test; ** represents significance (*p* < 0.005).

**Table 6:** Antioxidant activity of inulin using ABTS and DPPH assays, represented as mean ± standard deviation (sample in triplicate).

Mean/Standard Deviation	ABTS (μmol TE/g)	DPPH (μmol TE/g)
**Inulin**	262.5728 ± 34.74	9.092518 ± 10.43

**Table 7:** Phenolic compound contents of samples (n = 4) (mg gallic acid/g sample).

Parameters	Samples				
	Control	MI	MIC	MIM	ANOVA (*p* Value)
**Folin (mg gallic acid/g sample)**	2.35 ± 0.03	3.28 ± 0.22	11.03 ± 0.37	2.71 ± 0.02	*p* = 0.0000

Control: basic Chilean muffin; MI: muffin with inulin as fat substitute; MIC: muffin with inulin as fat substitute + cacao; MIM: muffin with inulin as fat substitute + Moringa.

**Table 8:** Folate vitamers (Folic Acid, THF, 5M-THF, and 5F-THF expressed as µg/100 g FW) and total folate (expressed as µg folic acid equivalents/100 g FW). (n = 4).

Samples (n = 4)	Folic Acid	THF	5M-THF	5F-THF	Total
**Control**	18.98 ± 1.52	148.00 ± 10.15	70.97 ± 0.16	374.27 ± 2.85	612.22 ± 5.95
**MI**	34.46 ± 1.25	8.43 ± 1.83	73.16 ± 1.11	172.20 ± 3.87	288.25 ± 3.33
**MIC**	54.39 ± 0.98	135.85 ± 4.01	103.57 ± 1.32	574.97 ± 23.98	868.77 ± 30.29
**MIM**	36.19 ± 2.81	68.76 ± 0.27	86.45 ± 1.16	297.45 ± 7.47	488.85 ± 9.39
**ANOVA** **(*p* Value)**	*p* = 0.0000	*p* = 0.0000	*p* = 0.0000	*p* = 0.0000	*p* = 0.0000

THF: tetrahydrofolate; 5M-THF: 5-methyltetrahydrofolate; 5F-THF: 5-formyltetrahydrofolate. Control: basic Chilean muffin; MI: muffin with inulin as fat substitute; MIC: muffin with inulin as fat substitute + cacao; MIM: muffin with inulin as fat substitute + Moringa.

**Table 9:** A color assessment of the samples (n = 4) according to the coordinates of the Hunter color system.

Samples (n = 4)	Colorimetry				
	c	l	a	b	h
**Control**	29.12 ± 2.35	61.55 ± 2.11	4.70 ± 0.66	28.72 ± 2.30	80.74 ± 0.61
**MI**	28.30 ± 1.83	60.66 ± 0.85	5.70 ± 0.50	27.73 ± 1.79	78.37 ± 0.87
**MIC**	12.00 ± 1.63	27.87 ± 1.16	9.03 ± 0.93	7.88 ± 1.43	40.90 ± 2.45
**MIM**	26.57 ± 3.22	56.03 ± 2.09	3.33 ± 0.08	26.36 ± 3.26	82.71 ± 1.14
**ANOVA** **(*p* Value)**	*p* = 0.0001	*p* = 0.0000	*p* = 0.0000	*p* = 0.0000	*p* = 0.0000

l: lightness; a: green shades; b: yellow shades; h: saturation; c: color. Control: basic Chilean muffin; MI: muffin with inulin as fat substitute; MIC: muffin with inulin as fat substitute + cacao; MIM: muffin with inulin as fat substitute + Moringa.

**Table 10:** A descriptive analysis of the sensory evaluation performed by a group of people with Parkinson’s disease.

Samples	Appearance	Aroma	Texture	Taste	Overall Color	Purchase Intention	Overall Acceptability
	Average ± SD						
**Control**	4.00 ± 0.74	3.74 ± 0.86	3.32 ± 0.99	4.00 ± 0.92	4.09 ± 0.92	3.36 ± 1.26	3.95 ± 0.722
**MI**	4.04 ± 1.11	3.13 ± 1.25	3.39 ± 0.98	3.82 ± 1.11	4.09 ± 0.92	3.22 ± 1.44	3.61 ± 1.03
**MIC**	4.13 ± 0.87	3.48 ± 0.95	3.70 ± 0.88	3.64 ± 0.95	4.14 ± 0.91	3.59 ± 1.37	3.82 ± 1.14
**MIM**	4.26 ± 0.915	3.39 ± 1.11	3.21 ± 0.99	3.77 ± 0.87	4.26 ± 0.75	3.72 ± 1.08	3.82 ± 0.80

Control: basic Chilean muffin; MI: muffin with inulin as fat substitute; MIC: muffin with inulin as fat substitute + cacao; MIM: muffin with inulin as fat substitute + Moringa.

## Data Availability

The original contributions presented in the study are included in the article, further inquiries can be directed to the corresponding author/s.
